# Supplementary material for: A genetic driver of epileptic encephalopathy impairs gating of synaptic glycolysis
Source: bioRxiv. 2025 Jun 19:2025.06.17.660213. Preprint. [Version 1] doi: 10.1101/2025.06.17.660213 (PMC12224543; doi:10.1101/2025.06.17.660213)
Supplement: Supplement 1 [file NIHPP2025.06.17.660213v1-supplement-1.pdf]

bioRxiv preprint doi: https://doi.org/10.1101/2025.06.17.660213; this version posted June 19, 2025. The copyright holder has placed this preprint (which was not certified by peer review) in the Public Domain. It is no longer restricted by copyright. Anyone can legally share, reuse, remix, or adapt this material for any purpose without crediting the original authors.

Supplementary data

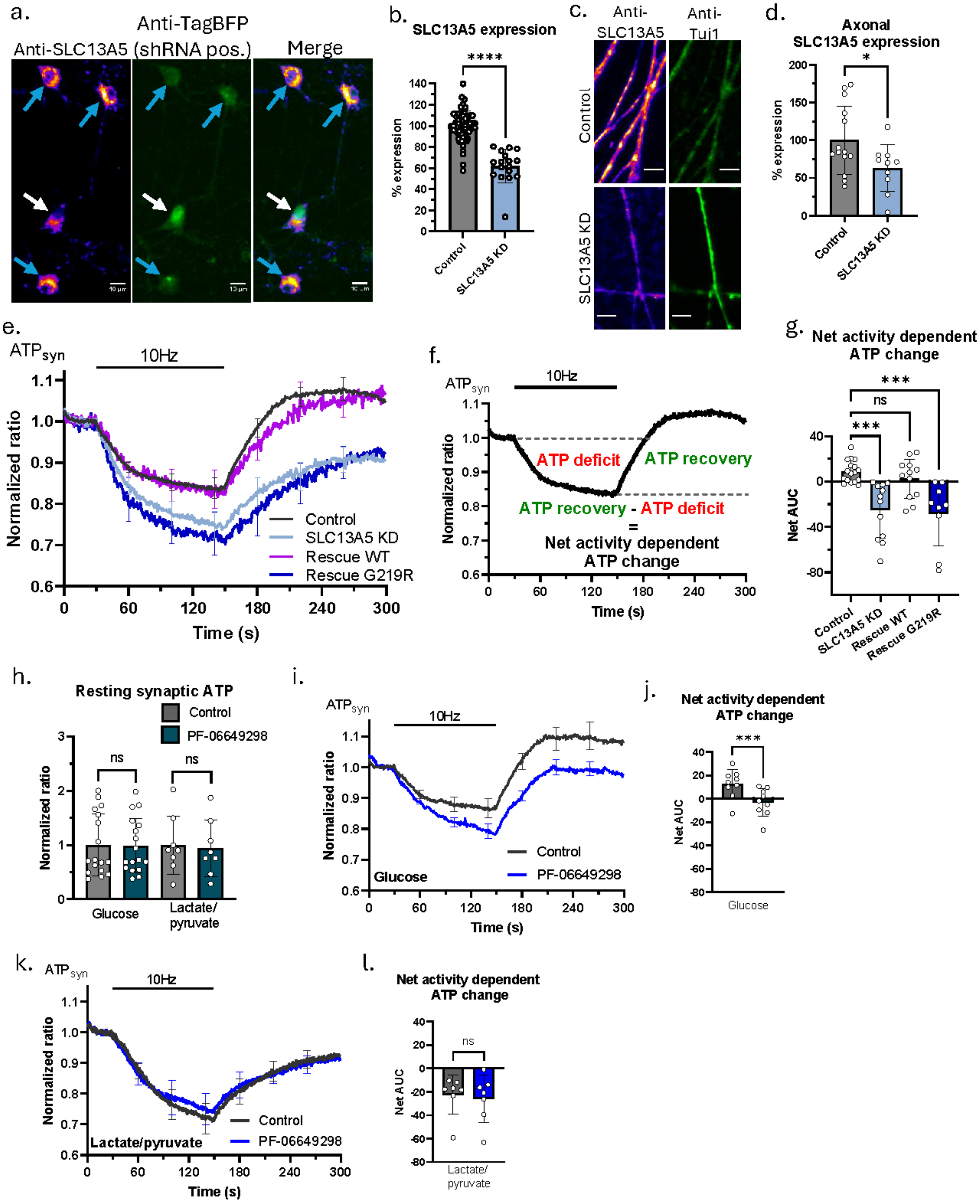

bioRxiv preprint doi: https://doi.org/10.1101/2025.06.17.660213; this version posted June 19, 2025. The copyright holder has placed this preprint (which was not certified by peer review) in the Public Domain. It is no longer restricted by copyright. Anyone can legally share, reuse, remix, or adapt this material for any purpose without crediting the original authors.

Figure S1. SLC13A5 is needed for activity dependent ATP production from glucose.
(a) Representative immunofluorescence images of control (blue arrows) and SLC13A5 KD (white arrow) hippocampal neurons stained with anti-SLC13A5 antibody (pseudo-color) and anti-TagBFP nanobody (green). Scale bar is 10 μm.
(b) Mean SLC13A5 expression +/-SEM in somas of control (n=65) and SLC13A5 KD (n=17) neurons.
(c) Representative immunofluorescence images of axons from control (upper) and SLC13A5 KD (lower) hippocampal neurons stained with anti-SLC13A5 (pseudo-color) and anti-Tuj1 (green) antibodies.
(d) Mean axonal SLC13A5 expression +/-SEM in control (n=14) and SLC13A5 KD (n=10) neurons.
(e) Average +/-SEM synapto-iATPSnFR2-miRFP670nano3 traces for control (black), SLC13A5 KD (light blue), rescue with WT SLC13A5 (magenta), and rescue with G219R mutant SLC13A5 (dark blue) neurons stimulated with 1200 APs at 10 Hz.
(f) Average control synapto-iATPSnFR2-miRFP670nano3 trace with markings showing AUC used for quantification of the net activity dependent ATP change.
(g) Mean presynaptic "net activity dependent ATP change" +/-SEM, quantified as shown in (f) for control (n=17), SLC13A5 KD (n=12), Rescue with WT SLC13A5 (n=12), and Rescue with G219R mutant of SLC13A5 (n=9) neurons.
(h) Mean resting synaptic ATP +/-SEM depicted as control-normalized iATPSnFR2-miRFP670nano3 ratio for control with glucose (n=17), PF-06649298 with glucose (n=17), control with lactate/pyruvate (n=8), and PF-06649298 with lactate/pyruvate (n=8).
(i) Average +/-SEM synapto-iATPSnFR2-miRFP670nano3 traces for control (black) and PF-06649298 treated (blue) neurons stimulated with 1200 APs at 10 Hz in glucose containing buffer.
(j) Mean net activity dependent ATP change +/-SEM, for control (n=9) and PF-06649298 treated (n=9) neurons in glucose containing buffer.
(k) Average +/-SEM synapto-iATPSnFR2-miRFP670nano3 traces for control (black) and PF-06649298 treated (blue) neurons stimulated with 1200 APs at 10 Hz in lactate/pyruvate containing buffer.
(l) Mean net activity dependent ATP change +/-SEM, for control (n=7) and PF-06649298 treated (n=7) neurons in lactate/pyruvate containing buffer.

bioRxiv preprint doi: https://doi.org/10.1101/2025.06.17.660213; this version posted June 19, 2025. The copyright holder has placed this preprint (which was not certified by peer review) in the Public Domain. It is no longer restricted by copyright. Anyone can legally share, reuse, remix, or adapt this material for any purpose without crediting the original authors.

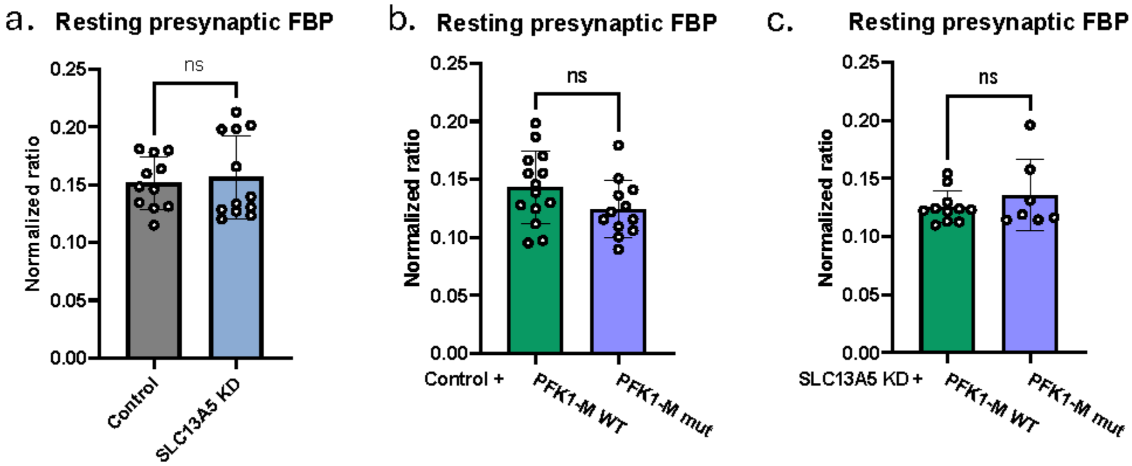

Figure S2. SLC13A5 KD and PFK1 OE don't affect resting presynaptic FBP levels.
(a-c) Mean resting presynaptic FBP +/-SEM, quantified as Hylight green-to-blue excitation ratio, for (a) control (n=11) and SLC13A5 KD (n=12), (b) control PFK1-M WT OE (n=14) and control PFK1-M mut OE (n=12), (c) SLC13A5 KD PFK1-M WT OE (n=11) and SLC13A5 PFK1-M mut OE (n=7) neurons.

bioRxiv preprint doi: https://doi.org/10.1101/2025.06.17.660213; this version posted June 19, 2025. The copyright holder has placed this preprint (which was not certified by peer review) in the Public Domain. It is no longer restricted by copyright. Anyone can legally share, reuse, remix, or adapt this material for any purpose without crediting the original authors.

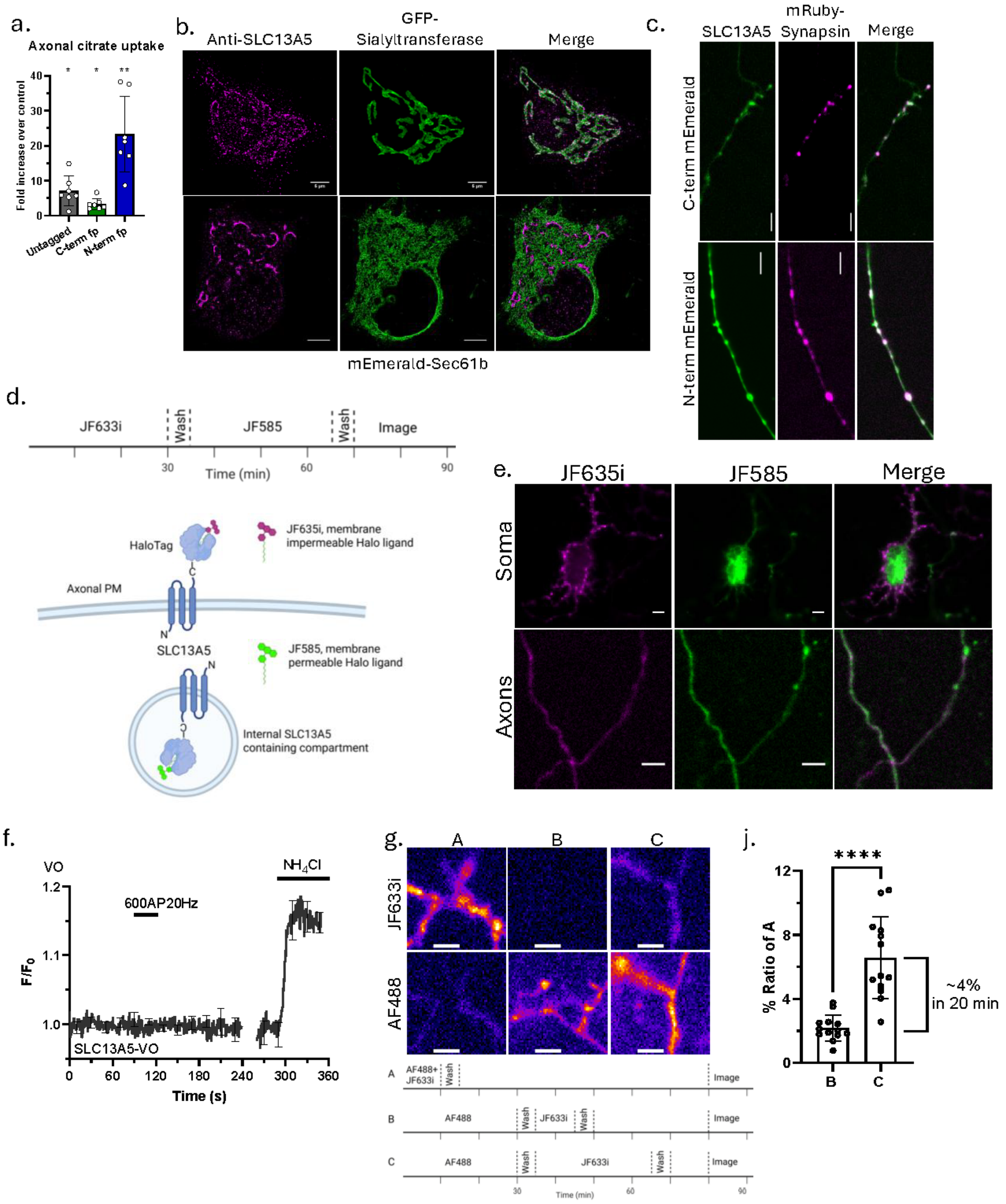

Figure S3. Intracellular SLC13A5 localization.

(a) Mean fold increase in axonal citrate uptake over control +/-SEM in response to 1 mM citrate superfusion, quantified for neurons overexpressing untagged (n=7), c-terminally tagged (n=7), and n-terminally tagged (n=7) SLC13A5 fusion constructs.

bioRxiv preprint doi: https://doi.org/10.1101/2025.06.17.660213; this version posted June 19, 2025. The copyright holder has placed this preprint (which was not certified by peer review) in the Public Domain. It is no longer restricted by copyright. Anyone can legally share, reuse, remix, or adapt this material for any purpose without crediting the original authors.

(b) Representative high-resolution Airyscan images of neuronal soma showing endogenous SLC13A5 staining (both left panels), GFP-Sialyltransferase (middle upper panel), mEmerald-Sec61b (middle lower panel) and merged images (right panels). Scale bar is 5 μm.
(c) Representative images of neurons expressing c-, and n-terminally mEmerald-tagged SLC13A5 fusion proteins co-transfected with mRuby-Synapsin. Scale bar is 5 μm.
(d) Experimental protocol (upper panel) and schematic illustration of predicted intracellular localization of SLC13A5-Halo construct and labelling with membrane impermeable (JF635i) and permeable (JF585) Halo-Tag ligands (lower panel).
(e) Representative images of neurons expressing SLC13A5-Halo construct and labelled according to the protocol illustrated in (d). Images show PM and intracellular localization of SLC13A5-Halo construct.
(f) Average +/-SEM VO trace in response to 600AP at 20 Hz and NH_4Cl superfusion.
(g) Representative images of neurons expressing SLC13A5-Halo construct labelled with membrane impermeable Halo-Tag dyes (JF635i and AF488) according to the protocol shown below. Scale bar is 4 μm.
(j) Analysis of the experiment shown in (g); JF635i to AF488 ratios of experimental conditions B and C were normalized as percentages of A, and reported as mean +/-SEM, n=13. The difference between ratios of C and B is 4%, while incubation time difference is 20 minutes, corresponding to 4% recycling in 20 minutes.

bioRxiv preprint doi: https://doi.org/10.1101/2025.06.17.660213; this version posted June 19, 2025. The copyright holder has placed this preprint (which was not certified by peer review) in the Public Domain. It is no longer restricted by copyright. Anyone can legally share, reuse, remix, or adapt this material for any purpose without crediting the original authors.

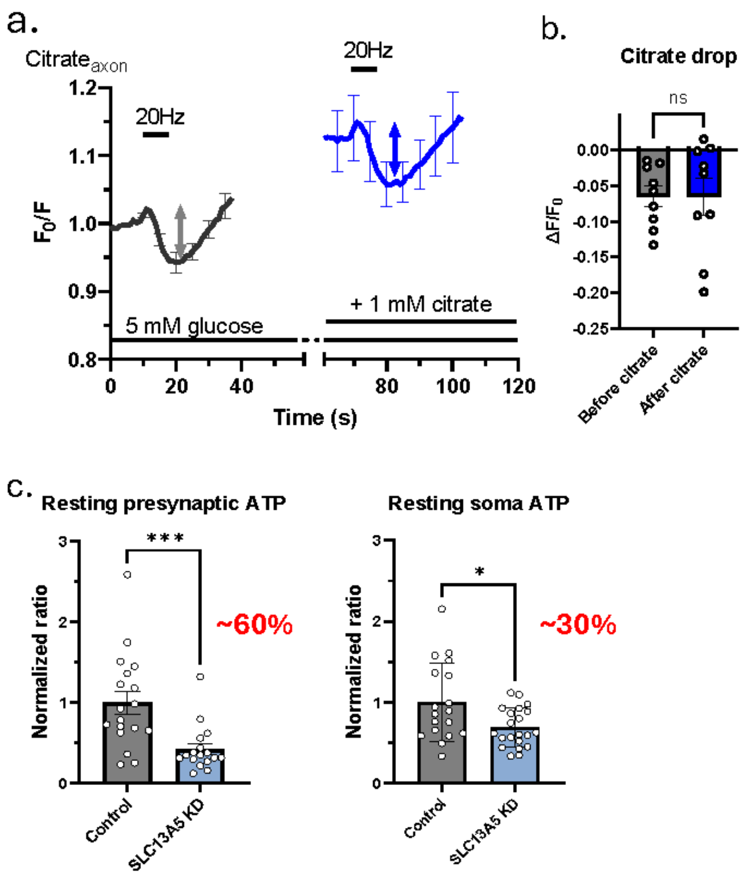

Figure S4. SLC13A5 preferentially functions in axons.
(a) Average +/-SEM axonal Citroff1 traces of control neurons before (black) and after 1 mM citrate superfusion (blue) in response to 100 APs at 20 Hz.
(b) Mean citrate drop +/-SEM in response to 100 APs at 20 Hz, quantified for neurons before (n=9) and after 1 mM citrate superfusion (n=9).
(c) Mean resting presynaptic (left) and soma (right) ATP +/-SEM, depicted as iATPSnFR2-miRFP670nano3 ratio, normalized to respective controls. Control presynaptic, n=18; SLC13A5 KD presynaptic, n=17; Control soma, n=18; SLC13A5 KD soma, n=22.

bioRxiv preprint doi: https://doi.org/10.1101/2025.06.17.660213; this version posted June 19, 2025. The copyright holder has placed this preprint (which was not certified by peer review) in the Public Domain. It is no longer restricted by copyright. Anyone can legally share, reuse, remix, or adapt this material for any purpose without crediting the original authors.

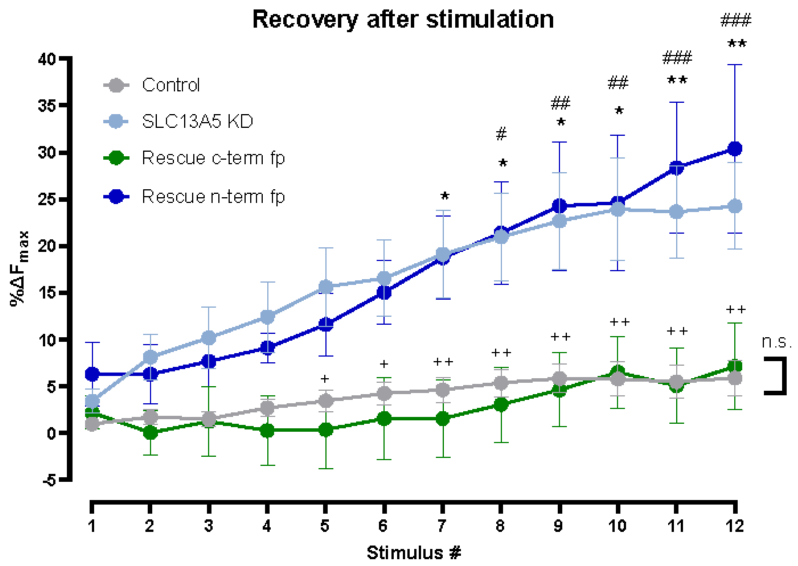

Figure S5. Proper SLC13A5 localization matters for synaptic function
Recovery after stimulation, quantified as remaining vG-pH fluorescence following AP train, for control (black, n=19), SLC13A5 KD (light blue, n=14), Rescue with c-terminally tagged SLC13A5 (green, n=7), and rescue with n-terminally tagged SLC13A5 (brown, n=17) neurons.
